# N-terminal Acetylation Levels Are Maintained During Acetyl-CoA Deficiency in Saccharomyces cerevisiae*[Fn FN2]

**DOI:** 10.1074/mcp.RA118.000982

**Published:** 2018-08-27

**Authors:** Sylvia Varland, Henriette Aksnes, Fedor Kryuchkov, Francis Impens, Delphi Van Haver, Veronique Jonckheere, Mathias Ziegler, Kris Gevaert, Petra Van Damme, Thomas Arnesen

**Affiliations:** From the ‡Department of Biomedicine, University of Bergen, N-5020 Bergen, Norway;; §Department of Biological Sciences, University of Bergen, N-5020 Bergen, Norway;; ¶Donnelly Center for Cellular and Bio‡molecular Research, University of Toronto, Toronto, ON M5S 3E1, Canada;; ‖VIB-UGent Center for Medical Biotechnology, B-9000 Ghent, Belgium;; **Department of Biomolecular Medicine, Ghent University, B-9000 Ghent, Belgium;; ‡‡VIB Proteomics Core, B-9000 Ghent, Belgium;; §§Department of Surgery, Haukeland University Hospital, N-5021 Bergen, Norway

**Keywords:** Acetylation, Molecular biology, N-terminal modifications, Tandem Mass Spectrometry, Yeast, Acetyl coenzyme A, Metabolism, N-terminal acetylation, N-terminomics, Ribosomal proteins

## Abstract

N-terminal acetylation (Nt-acetylation) is a highly abundant protein modification in eukaryotes and impacts a wide range of cellular processes, including protein quality control and stress tolerance. Despite its prevalence, the mechanisms regulating Nt-acetylation are still nebulous. Here, we present the first global study of Nt-acetylation in yeast cells as they progress to stationary phase in response to nutrient starvation. Surprisingly, we found that yeast cells maintain their global Nt-acetylation levels upon nutrient depletion, despite a marked decrease in acetyl-CoA levels. We further observed two distinct sets of protein N termini that display differential and opposing Nt-acetylation behavior upon nutrient starvation, indicating a dynamic process. The first protein cluster was enriched for annotated N termini showing increased Nt-acetylation in stationary phase compared with exponential growth phase. The second protein cluster was conversely enriched for alternative nonannotated N termini (*i.e.* N termini indicative of shorter N-terminal proteoforms) and, like histones, showed reduced acetylation levels in stationary phase when acetyl-CoA levels were low. Notably, the degree of Nt-acetylation of Pcl8, a negative regulator of glycogen biosynthesis and two components of the pre-ribosome complex (Rsa3 and Rpl7a) increased during starvation. Moreover, the steady-state levels of these proteins were regulated both by starvation and NatA activity. In summary, this study represents the first comprehensive analysis of metabolic regulation of Nt-acetylation and reveals that specific, rather than global, Nt-acetylation events are subject to metabolic regulation.

## Abstract (precis)

Nt-acetylation is a prevalent protein modification catalyzed by N-terminal acetyltransferases using acetyl-CoA as acetyl donor. Here, we performed a global analysis of Nt-acetylation in yeast following nutrient starvation. Contrary to histone acetylation, which is sensitive to acetyl-CoA levels, we demonstrate that Nt-acetylation remains largely unaffected to changes in cellular metabolism. We did, however, identify two protein groups that were differentially Nt-acetylated, one showing the same sensitivity to acetyl-CoA as histones. We propose that specific, rather than global, Nt-acetylation events are subject to metabolic regulation.

## Graphical Abstract

**Figure:**
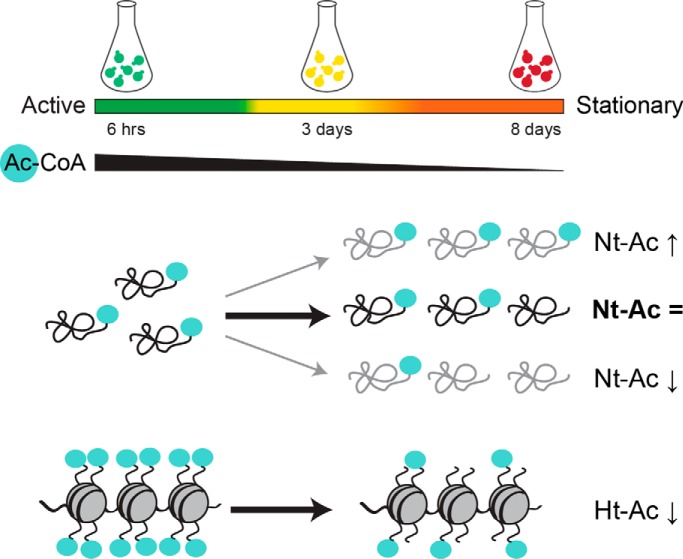

**Highlights**

- First global study on metabolic regulation of Nt-acetylation.
- Yeast cells maintain global Nt-acetylation levels during prolonged starvation.
- Nt-acetylation can in some cases be either up- or downregulated by starvation.
- Naa10/NatA affects the steady-state protein levels of Rsa3 and Rpl7a.

N-terminal acetylation (Nt^1^-acetylation) is a widespread protein modification, and ∼70% of all yeast protein species are fully or partially Nt-acetylated (1–4). The Nt-acetylation process involves the transfer of an acetyl moiety from acetyl-coenzyme A (acetyl-CoA) to the free α-amino group of (nascent) protein N termini. The N-terminal acetyl group affects various protein properties, including protein quality control and stability (5–7), protein folding (8–10), protein-protein interactions (11–13), and subcellular localization (11–14) as well as cellular processes such as cytoskeleton dynamics (15) and gene regulation (16). In yeast Nt-acetylation is catalyzed by five different N-terminal acetyltransferases (NATs), termed NatA to NatE. Each NAT is composed of one or more specific subunits and acetylates a distinct subset of proteins, primarily based on the nature of the two first amino acids (1).

Despite the recent developments in the field of Nt-acetylation, the regulatory mechanisms underlying this common protein modification remain poorly understood. Several findings suggest that Nt-acetylation plays a key role in cellular surveillance systems that counteract environmental stressors. In the plant *Arabidopsis thaliana* prolonged drought stress leads to an accumulation of the stress hormone abscisic acid, which causes a down-regulation of NatA. The diminished level of NatA and subsequent reduction of NatA-mediated Nt-acetylation elicits a stress response that limits plant growth and promotes plant survival (17). This study by Linster *et al.* was the first to describe a hormone-dependent adaption of NAT activity because of environmental stress. Moreover, Warnhoff and colleagues showed that reduced NAT activity contributes to increased resistance to heat, oxidative stress, and heavy metals in the nematode *Caenorhabditis elegans* (18). In yeast Nt-acetylation promotes nuclear-cytoplasmic shuttling of proteasomes during starvation (19). Nt-acetylation was also proved essential for development and viability of multicellular eukaryotes (17, 20–22). Taken together, these findings underscore the biological importance of Nt-acetylation and strongly suggest that it has a regulatory role in stress responses. Still, the molecular mechanisms at play and the protein-specific consequences of Nt-acetylation have only been elucidated for a limited number of substrates (2, 23).

Protein acetylation also frequently occurs at the ε-amino group of lysine residues within proteins (lysine acetylation or Nε-acetylation) in a post-translational process catalyzed by lysine acetyltransferases (KATs) (23, 24). In fact, reversible lysine acetylation of histone tails constitutes a major part of the histone code (25). The intermediate metabolite acetyl-CoA serves as acetyl donor for all known acetyltransferases, indicating a crosstalk between protein acetylation and cellular metabolism (24, 26, 27). Indeed, several studies have shown that fluctuations in acetyl-CoA availability can affect lysine acetylation (28–32). Inactivation of acetyl-CoA synthetase 2 (Acs2), which is the main supplier of nucleocytosolic acetyl-CoA in yeast, leads to a rapid decrease in histone acetylation and reduced global gene transcription (30). Furthermore, yeast cells display reduced histone acetylation following entry into the stationary phase (28). Friis and colleagues demonstrated that refeeding yeast cells in stationary phase can temporary restore histone acetylation levels. This effect is mainly mediated by the untargeted activity of Piccolo NuA4 and SAGA complexes and relies on glycolysis. Together these findings link cellular metabolism and quiescence to chromatin regulation (29). A correlation between glucose metabolism and histone acetylation was also reported in mammalian cells. Wellen *et al.* showed that knockdown of ATP citrate lyase (ACL), which is the primary source of cytosolic acetyl-CoA in mammalian cells, leads to a significant reduction in histone acetylation, whereas nonhistone targets, such as tubulin, appear unaffected (31). Like yeast, the addition of acetate rescued the effect in a manner dependent on AceCS1. Additionally, growth-arrested yeast cells show a global increase in mitochondrial lysine acetylation in a glycolysis-dependent manner (32).

The findings linking cellular metabolism to global histone acetylation (29–31) and the fact that many proteins are only partially Nt-acetylated (4) suggest that the NAT machinery might respond to metabolic cues. To address this hypothesis, we examined the effect of altered cellular metabolism on the global level of Nt-acetylation in the budding yeast *Saccharomyces cerevisiae*. More specifically, we compared the degree of Nt-acetylation between yeast cells in exponential growth phase and starvation-induced stationary phase using positional proteomics (N-terminal COFRADIC). To our surprise, the overall Nt-acetylation levels remained remarkably constant upon nutrient starvation causing acetyl-CoA depletion. Intriguingly however, Nt-acetylation appeared dynamic in some specific cases. In summary, our work provides an important insight into metabolic regulation of protein Nt-acetylation in yeast.

## EXPERIMENTAL PROCEDURES

### 

#### 

##### Yeast Strains

A complete list of yeast strains and their genotypes is given in supplemental Table S1, with the majority being derivatives of BY4741. Deletion strains were generated by PCR-based homologous recombination using *hphNT1* or *kanMX4* selection markers. Gene-specific markers were PCR amplified from *pFA6a-hphNT1* (33) or respective *kanMX4*-containing deletion strains (EUROSCARF, Oberursel, Germany). Transformations were performed according to Gietz and Schiestl (34) using 10 μg of purified PCR product. Antibiotic selection was achieved using 250 μg/L hygromycin B (Invitrogen) or 200 μg/L geneticin (Gibco). Gene deletions were confirmed by colony PCR using crude DNA extracts (35). The primers used in this study are listed in supplemental Table S2.

##### Culturing Conditions

Yeast strains were cultured in standard YPD (Sigma Y1375; 2% glucose, 1% yeast extract, and 2% bacteriological peptone) at 30 °C with 250 rpm shaking. 100 ml culture volumes in 1 L Erlenmeyer flasks were used for the COFRADIC and shotgun proteome analyses, whereas 10 ml culture volumes in 50 ml Falcon tubes were used for acetyl-CoA profiling and immunoblot analyses. Overnight cultures were diluted to OD_600_ ≈ 0.2. Cells in exponential growth phase were harvested after 6 h cultivation (OD_600_ 1.0–1.5), whereas cells in the semi-active and stationary growth phase were harvested after 3 and 8 days, respectively. Stationary cells were added 0% (control), 2% or 10% (w/v) of d-glucose and harvested after 24 h, resulting in 9 days of total cultivation time. Cell pellets were washed twice with cold MilliQ water and stored at −20 °C until further processing.

##### Acetyl-CoA Profiling by MS

Cell culturing was performed as described above and OD_600_ was measured prior to harvesting. Metabolites were extracted using a modified filter quench method originally described by Crutchfield *et al.* (36). Cell harvesting was achieved by vacuum-assisted filtration over 0.45 μm nylon membrane filters (Supelco). To quench cell metabolism and extract metabolites, the cell-containing filters were immediately placed in preheated extraction buffer (1 ml of 80% ethanol) and incubated for 3 min at 80 °C with 1400 rpm agitation. After the samples had cooled down on ice, they were transferred to new tubes and filters were washed 3 times with 340 μl ice-cold acetonitrile. Cell debris and other insoluble material was removed by centrifugation at 16,100 × *g* for 15 min at 4 °C and extracts were stored at −80 °C prior to LC-MS analysis. Targeted analysis of acetyl-CoA levels was achieved on a Dionex UltiMate 3000 Rapid Separation LC system coupled to a Q Exactive MS instrument (Thermo Fisher Scientific, Germany). Chromatographic separation was conducted using ACQUITY UPLC BEH Amide Column, (130Å, 1.7 μm, 2.1 mm × 50 mm (Waters Inc.) at flowrate of 0.4 ml/min. Solvent A consisted of 50 μm NH_4_HCO_3_ pH = 9.2 and acetonitrile was used as Solvent B. The metabolites were separated in a 6 min program with the following HPLC gradient conditions: 80% to 65% B at 0–4 min, 1% B from 4–4.8 min, and 80% B from 4.8–8 min. Injection volumes were normalized to cell content in extraction solvents. The following parameters of heated electrospray ion (HESI) source were used: Sheath gas flow rate: 50 units; Aux gas flow rate: 13 units; Sweep gas flow rate: 3 units; Spray voltage: 3.5 kV; Capillary temperature: 263 °C; S-lens RF level: 90; Aux gas heater temperature: 425 °C. Mass spectra were recorded in the positive ion electrospray ionization mode and in single ion monitoring (SIM) mode using the following acquisition parameters: Target-SIM, polarity: positive, Resolution: 140,000 at 200 *m*/*z*; AGC target: 1E5 ions, Maximum IT: 512 ms, Isolation window: 4.0 *m*/*z*, isolation offset: 1.0 *m*/*z*. The inclusion list consisted of the mass-to-charge ratio of the singly charged acetyl-CoA ion (810.13305 *m*/*z*). Data analysis was done using the label-free mass spectrometry differential analysis platform SIEVE Version 2.2.38 followed by manual inspection of LC-MS data. The following optional parameters of the SIEVE platform were used: Algorithm: FRAME, Experiment Target: METABOLOMICS, Experiment Type: NORMALIZED_TREND, ScanFilter: FTMS + p ESI SIM ms [809.13–813.13], AligmentMinIntensity: 10,000, MZWidthPPM: 5.

##### N-Terminal COFRADIC Analyses, LC-MS/MS Analysis, and Data Storage

Preparation of yeast cell extracts for N-terminal COFRADIC and subsequent LC-MS/MS analysis and data processing were performed as previously described (37). The analyses were performed on whole cell lysates from BY4742 obtained at 6 different metabolic conditions: yeast cultures grown for 6 h, 3 days, 8 days, 9 days + 0% glucose, 9 days + 2% glucose, and 9 days + 10% of glucose. All primary protein amines were blocked using an *N*-hydroxysuccinimide ester of (stable isotopic encoded) acetate at the protein level (*i.e.* an NHS ester of ^13^C_2_D_3_ acetate) to enable the assignment and the calculation of the degree of *in vivo* Nt-acetylation. In each case, a methionine oxidation step was introduced between the primary and secondary RP-HPLC separations, thereby shifting all methionine-containing N-terminal peptides to earlier elution times, allowing their enrichment (38). The obtained peptide mixtures were introduced into an LC-MS/MS system, the Ultimate 3000 (Dionex, Amsterdam, The Netherlands) in-line connected to an LTQ Orbitrap XL mass spectrometer (Thermo Fisher Scientific, Bremen, Germany) and LC-MS/MS analysis was performed as described previously (3, 4). The generated MS/MS peak lists were searched with Mascot using the Mascot Daemon interface (version 2.2.0, Matrix Science). Searches were performed in the Swiss-Prot database with taxonomy set to *Saccharomyces cerevisiae* (baker's yeast) (UniProtKB/Swiss-Prot database version 2016_01, containing 6,729 protein entries). ^13^C_2_D_3_-acetylation of lysine side-chains, carbamidomethylation of cysteine and methionine oxidation to methionine-sulfoxide were set as fixed modifications. Variable modifications were ^13^C_2_D_3_-acetylation and acetylation of protein N termini. Pyroglutamate formation of N-terminal glutamine was additionally set as a variable modification. Mass tolerance on precursor ions was set to 10 ppm (with Mascot's C13 option set to 1) and on fragment ions to 0.5 Da. Endoproteinase semi-Arg-C/P (Arg-C specificity with arginine-proline cleavage allowed) was set as enzyme, not allowing for missed cleavages. The peptide charge was set to 2+, 3+ and the instrument setting was put to ESI-TRAP. Only peptides that were ranked one, had a minimum amino acid length of seven and scored above the threshold score, set at 95% confidence, were withheld. The estimated false discovery rate by searching decoy databases (a shuffled version of the yeast Swiss-Prot database made by the DBToolkit algorithm (39)) was below 1% on the spectrum level and below 2% at the peptide level. Quantification of the degree of Nt-acetylation was performed as described (3). More specifically, acetylation *versus* heavy acetylation of N termini (Acetyl:2H(3)C13(2) N-term) was selected as quantification option, and carried out using the Mascot Distiller Quantitation Tool. All data management was done in ms_lims (40). The mass spectrometry proteomics data have been deposited to the ProteomeXchange Consortium (http://proteomecentral.proteomexchange.org) via the PRIDE partner repository (41) with the data set identifier PXD004326. Annotated spectra have been made available in MS-Viewer via ProteinProspector (http://prospector.ucsf.edu/prospector/mshome.htm) with the following search key 8i9cwelrjh.

##### Shotgun Proteome Analyses

Cell culturing was performed as described above. Pellets were washed with cold MilliQ and stored at −80 °C until further processing. Each condition was analyzed in triplicate and a total of 18 samples were prepared for LC-MS/MS analyses. Frozen cell pellets were dissolved in 1 ml lysis buffer (20 mm HEPES pH 8.0, 9 m urea) of which 500 μl was used further. Lysis was performed using FastPrep (MP Biomedicals) with Lysing Matrix Y beads. Samples were vortexed 7 × 10 s using the FastPrep device at 4 °C (one replicate sample was lost), followed by sonication with 3 pulses of 15 s at an amplitude of 20% using a 3 mm probe. After centrifugation for 15 min at 16,000 × *g* at room temperature (RT) to remove insoluble components, the protein concentration was measured and 500 μg of protein from each sample material was used further. Proteins were reduced by incubation with 5 mm DTT for 30 min at 55 °C and then alkylated with 10 mm iodoacetamide for 15 min at RT in the dark. Samples were further diluted with 20 mm HEPES pH 8.0 to a final urea concentration of 4 m and proteins were pre-digested with 2 μg LysC (Wako) (1/250, w/w) for 4 h at 37 °C. Samples were then further diluted to 2 m urea and digested with 5 μg trypsin (Promega) (1/100, w/w) overnight at 37 °C. The resulting peptide mixture was acidified by addition of 1% trifluoroacetic acid (TFA) and after 15 min incubation on ice, the samples were centrifuged for 15 min at 1780 × *g* at RT to remove insoluble components. Next, peptides were purified on SampliQ C18 columns (Agilent). These columns were first washed with 1 ml of acetonitrile and pre-equilibrated with 3 ml of solvent A (0.1% TFA in water/acetonitrile (98:2, v/v)) before samples were loaded on the column. After peptide binding, the column was washed with 2 ml of solvent A and peptides were eluted with 1.4 ml of solvent B (0.1% TFA in water/acetonitrile (20:80, v/v)). Purified peptides were dried completely by vacuum drying and stored at −20 °C until LC-MS/MS analysis. Purified peptides were re-dissolved in solvent A and ∼2 μg of each sample was injected for LC-MS/MS analysis on an Ultimate 3000 RSLCnano system (Thermo Fischer Scientific) in-line connected to a Q Exactive HF mass spectrometer (Thermo Fischer Scientific) equipped with a Nanospray Flex Ion source (Thermo Fischer Scientific). Trapping was performed at 10 μl/min for 4 min in solvent A on a 20 mm trapping column (made in-house, 100 μm internal diameter (I.D.), 5 μm beads, C18 Reprosil-HD, Dr. Maisch, Germany) after which the sample was loaded on a 400 mm analytical column (made in-house, 75 μm I.D., 1.9 μm beads C18 Reprosil-HD, Dr. Maisch). Peptides were eluted by a nonlinear solvent gradient from 2 to 56% solvent B' (0.1% formic acid (FA) in water/acetonitrile (2:8, v/v)) over 140 min at a constant flow rate of 250 nl/min, followed by a 15 min wash reaching 99% solvent B′ and re-equilibration with solvent A′ (0.1% FA in water/acetonitrile (2:8, v/v)). The column temperature was kept constant at 50 °C in a column oven (CoControl 3.3.05, Sonation). The mass spectrometer was operated in data-dependent mode, automatically switching between MS and MS/MS acquisition for the 16 most abundant ion peaks per MS spectrum. Full-scan MS spectra (375–1500 *m*/*z*) were acquired at a resolution of 60,000 in the Orbitrap analyzer after accumulation to a target value of 3,000,000. The 16 most intense ions above a threshold value of 22,000 were isolated (window of 1.5 Th) for fragmentation at a normalized collision energy of 28% after filling the trap at a target value of 100,000 for maximum 45 ms. MS/MS spectra (200–2000 m/z) were acquired at a resolution of 15,000 in the Orbitrap analyzer. The S-lens RF level was set at 55 and we excluded precursor ions with single and with unassigned charge states and with charge states above 5 from fragmentation selection. Data analysis was performed with MaxQuant (version 1.5.6.5) using the Andromeda search engine with default search settings including a false discovery rate set at 1% on both the peptide and protein level. Spectra were searched against the yeast proteins in the UniProt/Swiss-Prot database (database release version of August 2016 containing 6,721 yeast protein sequences, www.uniprot.org). The mass tolerance for precursor and fragment ions was set to 4.5 and 20 ppm, respectively, during the main search. Enzyme specificity was set as C-terminal to arginine and lysine, also allowing cleavage at proline bonds with a maximum of two missed cleavages. Variable modifications were set to oxidation of methionine residues and acetylation of protein N termini, whereas carbamidomethylation of cysteine residues was set as fixed modification. Matching between runs was enabled with a matching time window of 1 min and an alignment time window of 20 min. Only proteins with at least one unique or razor peptide were retained leading to the identification of 2,707 yeast proteins. Proteins were quantified by the MaxLFQ algorithm integrated in the MaxQuant software. A minimum ratio count of two unique or razor peptides was required for quantification. Further data analysis was performed with the Perseus software (version 1.5.5.3) after loading the protein groups file from MaxQuant. Proteins only identified by site, reverse database hits and potential contaminants were removed, and replicate samples were grouped. Proteins with less than three valid values in at least one group were removed and missing values were imputed from a normal distribution around the detection limit leading to a list of 1,918 quantified proteins that was used for further data analysis. The shotgun proteomics data have been deposited to the ProteomeXchange Consortium (http://proteomecentral.proteomexchange.org) via the PRIDE partner repository (41) with the dataset identifier PXD009214. Annotated spectra have been made available in MS-Viewer via ProteinProspector (http://prospector.ucsf.edu/prospector/mshome.htm) with the following search key ibi5m5495s.

##### Immunoblot Analysis

Protein extracts from yeast cells were prepared according to Kushnirov (42) with minor adjustments. Alkaline treated cell pellets were resuspended in sample buffer (60 mm Tris-HCl, pH 6.8, 5% glycerol, 2% SDS, 100 mm DTT, and bromphenol blue) to a final concentration of 50–75 mg wet weight pellet per ml. The cell suspensions were subsequently incubated at 90 °C for 10 min. To remove cell debris, the suspensions were centrifuged at 10,000 × *g* for 30 s. Equal amounts of protein extract (0.25 - 0.5 mg of wet weight) were resolved by Mini-PROTEAN TGX stain-free protein gels (Bio-Rad) and transferred onto a nitrocellulose membrane (GE Healthcare). Histone H4 was detected with mouse anti-Histone H4 and anti-acetyl-Histone H4 (Merck Millipore, 05–858 and 06–598, respectively) using a 1:1000 dilution. TAP-tagged proteins were detected with rabbit anti-TAP (Pierce, CAB1001) at 1:500–1:6000 dilutions. Actin served as loading control and was probed with mouse anti-beta-actin (Abcam, ab8224) at 1:4000–1:6000 dilutions. Membranes were probed with horseradish peroxidase-linked secondary antibodies (GE Healthcare) at 1:4000–1:6000 dilutions. All antibodies were diluted in milk-based blocking buffer (1–5% (w/v) dry milk powder, 1 × PBS, 0.5% (w/v) Tween-20). The immunoblots were incubated with SuperSignal West Pico (Thermo Scientific) and chemiluminescence signals were detected using ChemiDoc XRS+ and imaged with ImageLab v5.1 Beta (both from Bio-Rad). Immunoblots shown are representative of at least three independent experiments. Densitometry analysis was performed using ImageLab

##### Experimental Design and Statistical Rationale

Acetyl-CoA profiling was performed using six biological samples with six technical replicates. Significance was determined by Tukey's multiple comparisons test where *p* values <0.0001 (****) and <0.05 (*) were considered significant. Abundance measurements were normalized to average absolute abundance at 6 h. Mean ± S.D. is shown as a line. The COFRADIC analyses were performed with one biological sample for each condition. Differential Nt-acetylation was here defined as a minimum shift of 10% in Nt-acetylation between active phase and semi-active and/or stationary phase. This cut-off value was based on the very high correlation between the degrees of Nt-acetylation calculated in independent N-terminal proteomics experiments (R^2^ = 0.998), for which a *p* value of *p* ≤ 0.01 was calculated when considering a minimum shift of 10% (3). Shotgun proteomics experiments were performed in biological triplicates except for day 9 with 10% of glucose where one sample was omitted because of a technical error (see above). A two-way ANOVA test was performed to compare the intensities of the proteins in the time group with the glucose percentage group. Proteins with *p* value <0.01 in at least one of both groups were significantly regulated. Gene Ontology (GO) enrichment analysis was performed using the GO Slim Mapper from the Saccharomyces Genome Database (SGD) using the quantified proteins as background set. *p* values were calculated using Fisher's exact test. GO terms with *p* value < 0.01 were considered statistically significant. Immunoblots shown are representative results of at least three independent biological experiments. The densitrometric values refer to the experiment shown.

## RESULTS

### 

#### 

##### Histone Acetylation is Sensitive to Acetyl-CoA Levels

**Fig. 1.**
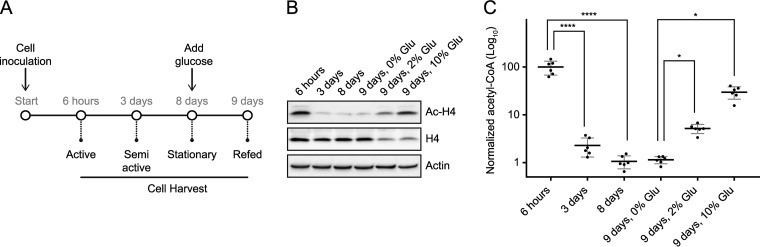
**Nutrient availability affects histone acetylation and cellular acetyl-CoA levels.** *A*, Schematic outline of the experimental setup. Yeast cells were grown in rich medium and harvested at the indicated growth phases. Stationary cells were given 0%, 2% or 10% (w/v) of glucose and harvested after an additional 24 h of growth. *B*, Histone acetylation declines as yeast cells progress to stationary phase, but is partially restored after glucose addition. Immunoblot analysis of H4 acetylation, bulk H4 expression, and actin (loading control) at indicated time points. *C*, Intracellular acetyl-CoA levels changes with nutrient availability. Metabolites were extracted from whole yeast cells at the indicated growth phases and acetyl-CoA levels were measured by MS. Data were normalized to the average value at 6 h. Normalized data are shown as log10. Mean ± S.D. is shown as lines. 6 h, 100%; 3 days, 2%; 8 days, 1%, 9 days + 0% glucose, 1%; 9 days + 2% glucose, 5%; 9 days + 10% glucose, 30%. ****, *p* < 0.0001; *, *p* < 0.05; *n* = 6.

Previous research has shown that histone acetylation is sensitive to acetyl-CoA availability (28–31), indicating that protein acetylation in general could be dynamic with respect to cellular metabolism. To investigate the effect of nutrient availability on global Nt-acetylation levels, yeast cells were grown in rich medium and harvested in exponential active, semi-active, and stationary growth phase (Fig. 1*A*). In addition, stationary cells were given 0%, 2% or 10% of glucose and harvested after an additional 24 h of cultivation. We validated the experimental setup with respect to histone acetylation using immunoblot analysis, confirming hypoacetylation of histone H4 following entry into stationary phase (Fig. 1*B*) (29). Glucose addition to stationary cells reversed the effect of nutrient starvation causing hyperacetylation of histone H4. Moreover, we performed metabolite extraction from whole yeast cells to monitor the level of acetyl-CoA. The intracellular level of acetyl-CoA was significantly reduced (*p* < 0.0001) when the cells entered the stationary phase (Fig. 1*C* and supplemental Table S3), associated with glucose exhaustion. In agreement with previous studies, the cellular level of acetyl-CoA was partly restored after glucose addition (29, 32, 43).

##### Nt-Acetylation Remains Generally Stable as Yeast Cells Progress to Stationary Phase

**Fig. 2.**
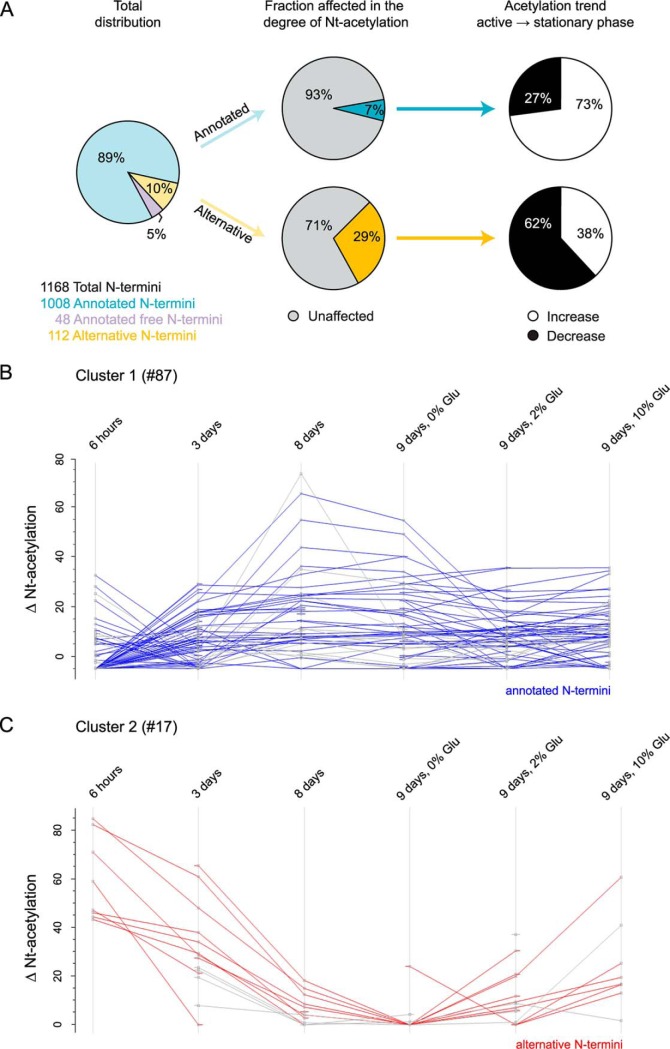
**Metabolic effect on Nt-acetylation in yeast.** *A*, Total distribution of annotated and alternative N termini identified and overall acetylation trend following prolonged starvation. Free N termini refers to N termini that cannot be acetylated (MP- and P- starting). (*B-C*) Differences in Nt-acetylation of affected N termini upon entry into stationary phase and following glucose addition. *k*-means clustering of affected N termini (104) revealed two distinct clusters (*p* = 0.000167). *B*, Cluster 1 is enriched for annotated N termini (blue) that are more Nt-acetylated in stationary phase and experiences a reduction of Nt-acetylation upon glucose addition. *C*, Cluster 2 is enriched for alternative N termini (red) and show an acetylation trend that is opposite of cluster 1.

Based on the observed fluctuations in histone acetylation, we hypothesized that yeast cells transitioning from exponential growth to stationary phase, which entails a significant reduction in acetyl-CoA levels (Fig. 1*C*), would experience a reduction in overall Nt-acetylation. To address this hypothesis, we used N-terminal COFRADIC to quantify Nt-acetylation in whole protein extracts from 6 different cellular conditions (Fig. 1*A*). In total, we identified 1,168 unique N termini (supplemental Table S4). Annotated N termini (*i.e.* N termini starting at position 1 or 2 of the protein sequence) constituted the majority (1,056) of identified N termini, whereas the remaining part (112) consisted of so-called alternative N termini (*i.e.* N termini starting beyond position 2 in the protein sequence). The latter likely point to alternative Nt-proteoforms generated by alternative translation initiation and/or splicing events (44). To our surprise, we found that the steady-state levels of Nt-acetylation of database annotated N termini remained fairly stable following entry into stationary phase, despite a prominent decrease in acetyl-CoA and histone acetylation levels (Fig 2*A*). In fact, the majority of identified N termini (∼90%) did not respond to major changes in cellular metabolism induced by prolonged starvation or the subsequent addition of glucose. Differential Nt-acetylation was here defined as a minimum shift of 10% in Nt-acetylation between exponential growth phase and semi-active and/or stationary growth phase (see Experimental Design and Statistical Analysis).

##### A Minor Fraction of The Yeast Proteome is Differentially Nt-Acetylated

Further analysis of the COFRADIC data revealed that the acetylation status of 104 N termini differed between exponential growth phase and intermediate or stationary phase (Fig. 2*A*), of which 71 N termini were database annotated (supplemental Table S5) and 33 were alternative N termini (supplemental Table S6). Based on generic *k*-means clustering of the differentially acetylated N termini, two clusters appeared as determined by a Chi-square test of independence (*p* = 0.000167). Cluster 1 was significantly enriched for database annotated N termini and displayed increased level of Nt-acetylation following nutrient starvation, an effect that in most cases was reversed by glucose addition (Fig. 2*B*). For example, the degree of Nt-acetylation of Smy2 (MI-) increased from 34% in exponential growth phase to 100% in stationary phase, and declined to 57% after glucose addition (Table I). Cluster 2, on the other hand, was enriched for alternative N termini (Fig. 2*C*) and presented the same acetylation trend as histones, becoming less acetylated following entry into stationary phase. For instance, the alternative N terminus of Eis1 (ME- at position 164) was 48% Nt-acetylated during exponential phase, but only 4% Nt-acetylated in stationary phase (Table I). Moreover, the Nt-acetylation level of Eis1 increased to 16% after glucose addition. In total, 73% of the affected annotated N termini were more Nt-acetylated in stationary phase (low acetyl-CoA) as compared with exponential phase (high acetyl-CoA) (Fig. 2*A*). Conversely, entry into the stationary phase caused a decrease in Nt-acetylation for a significant portion of the affected alternative N termini. Glucose addition reversed the effect for most of the affected N termini. Based on these observations, we suggest that specific, rather than global, Nt-acetylation events are subject to metabolic regulation.

**Table I:** Selected N-termini that are subject to differential Nt-acetylation upon entry into stationary phase

Standard name	Systematic name	N'term	Start	N-terminal acetylation in percentage						Cluster
				6 hrs	3 days	8 days	Day 9^a^			
							0%	2%	10%	
Annotated N-termini										
Atg8	YBL078C	MKSTF	1	26	28	65	63	44	38	ø
Atg22	YCL038C	SYGTI	2	-	60	-	55	58	69	-
Pbp1	YGR178C	MKGNF	1	72	93	100	100	94	92	5
Pcl8	YPL219W	ANDQD	2	74	92	-	100	86	-	-
Rpl7a	YGL076C	AAEKI	2	12	44	43	47	50	-	5
Rsa3	YLR221C	SAGDI	2	42	72	-	70	71	-	-
Smy2	YBR172C	MIAPD	1	34	59	100	90	53	57	ø
Tom70	YNL121C	MKSFI	1	18	47	46	50	56	56	ø
Tps1	YBR126C	TTDNA	2	34	44	46	48	49	56	ø
Tps2	YDR074W	TTTAQ	2	79	86	90	88	89	91	2
Alternative N-termini										
Eis1	YMR031C	METTS	164	48	38	13	4	16	-	
Fbp1	YLR377C	MEQAG	300	-	6	34	21	-	-	
Pgi1	YBR196C	MESNG	366	-	86	39	21	41	82	

^a^Stationary cells (day 8) were given 2% or 10% (w/v) glucose and harvested after additional 24 hours of growth. Hyphen (-) indicates that the N-terminus was not detected in the particular sample. Ø denotes samples that were detected and quantified by shotgun proteomics, but whose expression level was not significantly affected between the different conditions. In general, proteins in cluster 2 showed higher expression in stationary phase whereas proteins in cluster 5 were less expressed in stationary phase.

##### Differential Nt-Acetylation May in Some Cases Be Related to Changes in Protein Abundance

**Fig. 3.**
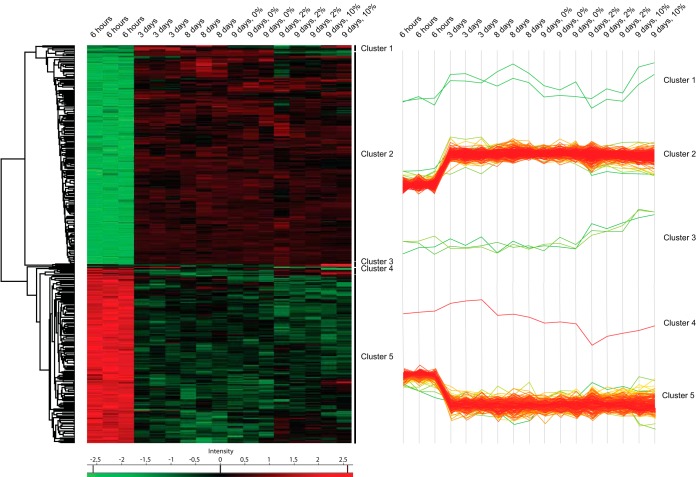
**Heat map of the nonsupervised hierarchical clustering of 401 yeast proteins that display differential expression levels across different metabolic conditions.** Each row represents a protein and metabolic conditions are depicted in columns (6 h, 3 days, 8 days, 9 days + 0% glucose, 9 days + 2% glucose, and 9 days + 10% glucose). The color key indicates relative abundance, ranging from green (low abundance) to red (high abundance). Five main clusters were observed when the significance threshold was set at *p* < 0.01. Three independent biological samples were obtained at each sampling time (except for 9 days + 10% glucose having only two).

Because COFRADIC analyses can only be used to determine changes in Nt-acetylation levels and not changes in overall protein abundance, we next performed label-free shotgun proteomics to quantify protein expression using the same cellular conditions. In total, we identified 2,707 proteins (supplemental Table S7) of which 1918 were reliably quantified (proteins with at least 3 valid LFQ intensity values in one of the six conditions, see Experimental Procedure) (supplemental Table S8). To reveal proteins whose expression level was significantly affected between the different metabolic conditions, sample groups were defined based on time (6 h *versus* 3 days *versus* 8 days *versus* 9 days) and glucose percentage (0% *versus* 2% *versus* 10%) and a two-way ANOVA test was performed to compare the intensities of the proteins in the time group with the glucose percentage group. For each protein, this resulted in a -log *p* value for time and a *p* value for glucose percentage. 401 proteins with a *p* value <0.01 in at least one of both groups were considered to be significantly regulated. The intensities of these proteins are shown in a heat map after nonsupervised hierarchical row clustering (Fig. 3). In the row tree, 5 clusters were observed, corresponding to proteins with different expression profiles in the sample groups (supplemental Table S9). In total, six proteins (Mxr1, Sse1, Met6, Gdh1, Ser3, and Glt1) were assigned to cluster 1, cluster 3, and cluster 4. In these cases, we observed varying expression changes upon entry into stationary phase, but they all showed increased protein abundance after glucose addition. Proteins in cluster 2 (218 proteins) exhibited increased expression in stationary phase whereas the opposite was the case for proteins in cluster 5 (177 proteins). For example, we observed increased expression of Fbp1 (Fructose-1,6-bisphosphatase; required for glucose metabolism) upon entry into stationary phase followed by a prominent decrease after glucose addition. Conversely, the protein level of Gpp1 (glycerol-1-phosphatase; involved in glycerol biosynthesis) decreased from exponential growth phase to stationary phase. The effect of prolonged starvation was partly reversed upon glucose addition. A gene ontology (GO) enrichment analysis of the proteins in cluster 2 and cluster 5 revealed a strong enrichment for genes involved in oxidation-reduction process (*p* value < 2.18e-14) and translation (*p* value < 1.13e-34), respectively. When glucose becomes exhausted from the growth medium yeast cells redirect their cellular metabolism from fermentation to respiration. This metabolic reprogramming is accompanied by reduced gene expression and protein translation. Hence, our findings agree with known proteome changes in response to prolonged growth (45).

We next compared the N-terminal COFRADIC data with the shotgun results to determine whether the observed differential Nt-acetylation was related to changes in protein abundance. 64 out of the 101 unique proteins that were found differentially Nt-acetylated were detected and quantified by label-free shotgun proteomics. Of these, 69% displayed protein abundance levels that were not significantly affected by changes in cellular metabolism (Table I and supplemental Table S10). We do note, however, that only 21% of the quantified proteins were differentially expressed, as opposed to 31% of the differentially Nt-acetylated proteins. For example, Cox12 (A-) showed a marked increase in Nt-acetylation following entry intro stationary phase (from 46% to 82%), but the Nt-acetylation level decreased to 36% following glucose addition. The expression level of Cox12 was relatively low in exponential growth phase, but rapidly increased as the yeast cells progressed to stationary phase (assigned to cluster 2). Thus, because we only looked at changes in the steady-state protein levels and not new protein synthesis, we cannot rule out the possibility that altered translation rates have an impact on the differential Nt-acetylation observed, especially for suboptimal NAT substrates.

##### Differential Nt-Acetylation Does Not Appear to Be Caused by NAT Enzyme Regulation

**Fig. 4.**
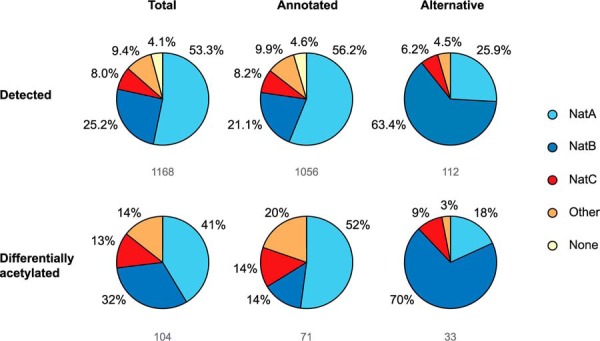
**Total distribution of differentially acetylated N termini among NAT substrate classes.** Differentially acetylated N termini were classified according to NAT specificity. The pie charts illustrate NAT distribution among total N termini identified and corresponding annotated and alternative N termini. The respective numbers are indicated below the pie charts. N termini which can be acetylated by several NATs are categorized as 'Other'. MP- and P-starting peptides cannot be acetylated and are classified as 'None'.

Yeast cells express five different NATs (NatA to NatE) that differ in their subunit composition and substrate specificity, which is mainly dictated by the nature of the first two amino acids of the substrate protein (1). We next compared the distribution of the affected N termini against the substrate profiles of the different NATs. Among the major NATs, NatA targets N termini which have gone through initiator methionine (iMet) removal, whereas NatB and NatC acetylate the iMet when followed by an acidic or a hydrophobic/amphiphatic residue, respectively (1). All the identified and differentially acetylated N termini were accordingly classified as NatA, NatB, or NatC-type substrates. N termini that could potentially be acetylated by several NATs (redundant) or are not acetylated at all (proline starting) (46) were classified as “other” or “none,” respectively. We found that all the major NAT substrate classes were represented among the N termini that were differentially acetylated (Fig. 4). More specifically, the annotated N termini within the NatC and other groups were most susceptible to differential Nt-acetylation (*i.e.* a 1.63 and 1.89 fold increase was observed, respectively) compared with NatB-type N termini. With respect to alternative N termini, we found that the NatB and NatC groups were more frequently affected in their degree of Nt-acetylation. Furthermore, classifying the differentially Nt-acetylated proteins according to increased or reduced degree of Nt-acetylation did not reveal any clear signs of up- or downregulation of specific NAT activity (data not shown). That is, the proteins that are subject to changes in their Nt-acetylation status do not share any particular NAT substrate profile but appear enriched for suboptimal NAT substrates.

**Fig. 5.**
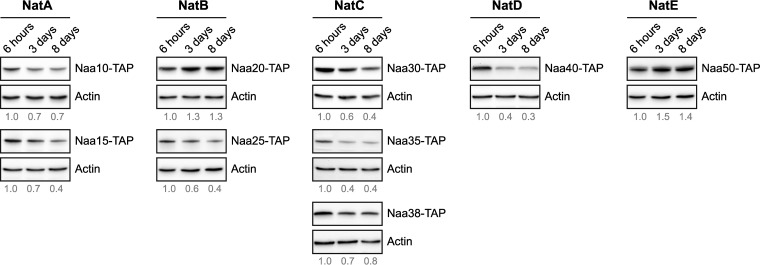
**Protein expression profiles of NAT subunits upon entry into stationary phase.** Yeast cells endogenously expressing a TAP-tagged NAT subunit were grown in rich medium and harvested at indicated time points. Whole cell extracts were analyzed by immunoblotting using anti-TAP and anti-actin (loading control). The proteins are listed according to NAT affiliation and the catalytic subunit is shown at the top. The protein level of each NAT subunit relative to actin at each time point were quantified by densitometry analysis and normalized to the value at 6 h.

To investigate the possibility that differential Nt-acetylation could be affected by altered NAT expression, we examined the protein levels of the individual NAT subunits by immunoblotting. Yeast cells endogenously expressing TAP-tagged NAT subunits were harvested at exponential, semi-active, and stationary growth phase. Generally, the protein levels of the individual NAT subunits decreased upon entry into stationary phase (Fig. 5). The most pronounced reduction of a catalytic subunit was observed for NatD (Naa40). In contrast, prolonged starvation caused increased levels of the catalytic components of NatB (Naa20) and NatE (Naa50). Although increased presence of Naa20 and Naa50 might explain why some N termini are more acetylated during starvation, the changes in NAT subunit expression levels *per se* do not provide a valid explanation for observed changes in the Nt-acetylome following nutrient starvation.

##### Protein-Specific Consequences of Differential Nt-Acetylation

**Fig. 6.**
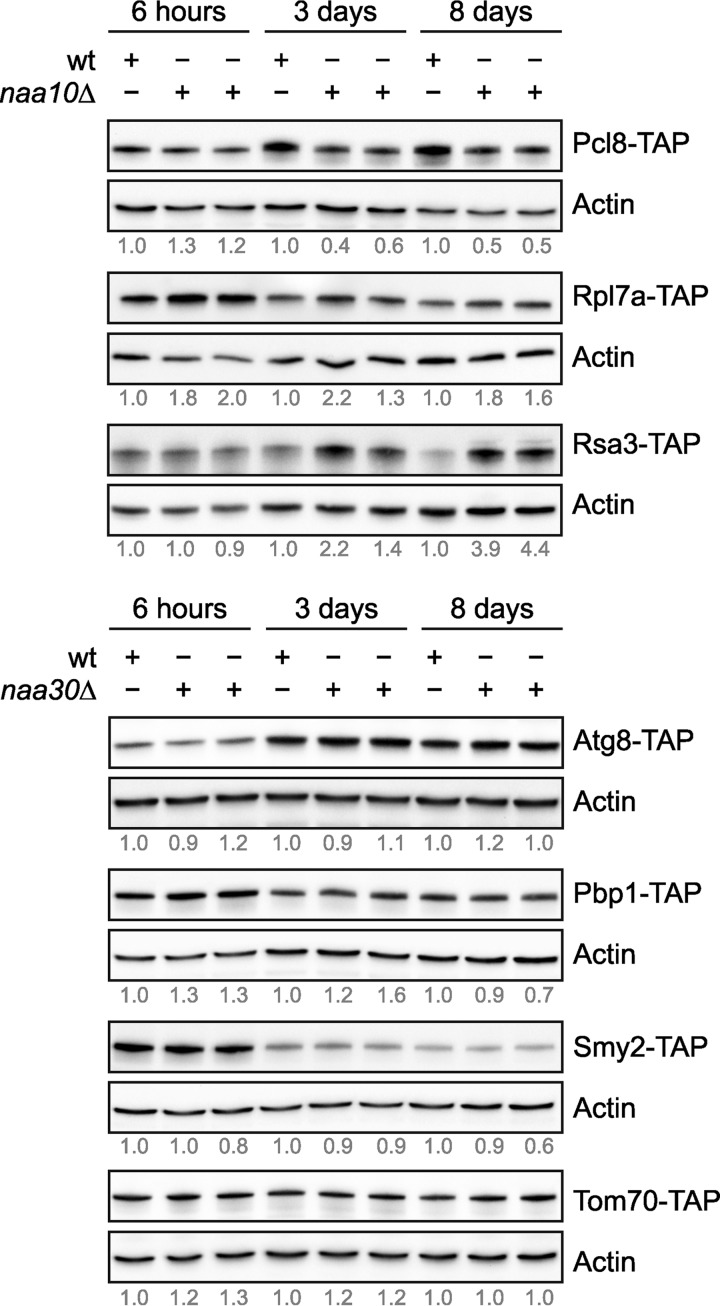
**Immunoblot analysis of differentially Nt-acetylated proteins under different metabolic conditions and in presence/absence of the cognate NAT.** Wild-type, *naa10*Δ and *naa30*Δ yeast strains endogenously expressing a unique TAP-tagged protein were grown in rich medium and harvested at indicated time points. Whole cell extracts were analyzed by immunoblotting using anti-TAP and anti-actin (loading control). Two individual deletion strains per candidate protein were used. The levels of TAP-tagged candidate proteins relative to actin at each time point were quantified by densitometry analysis and normalized to the wild-type sample within the same metabolic condition.

Finally, we examined whether differential Nt-acetylation affects the expression level of substrate proteins. We therefore selected, based on protein abundance and strain availability, seven proteins (Atg8, Pbp1, Pcl8, Rpl7a, Rsa3, Smy2, and Tom70) for further analyses. To this end, we used yeast strains endogenously expressing TAP-tagged candidate proteins and deleted the cognate catalytic NAT subunit by homologous recombination. This allowed us to study the expression level of Nt-acetylated and presumed unacetylated forms of the candidate proteins by immunoblot analyses. By comparing the steady-state protein levels in the wild-type strain and the deletion strain, we observed a substantial difference in the protein level of three confirmed NatA substrates (37) following entry into stationary phase (Fig. 6). The Nt-acetylation level of cyclin Pcl8 (A-), which negatively regulates glycogen synthesis (47), was found to be 73% Nt-acetylated in exponential growth phase (supplemental Table S4). The protein level of Pcl8 increased upon entry into stationary phase and simultaneously the protein became fully Nt-acetylated (100%). Interestingly, deletion of *NAA10* (*ARD1*) abolished the observed increase in Pcl8 protein levels during starvation. Overall, these findings suggest that NatA-mediated Nt-acetylation may directly or indirectly upregulate or stabilize Pcl8 during starvation. Increased Nt-acetylation after starvation was also observed for two components of the pre-ribosome complex (48, 49). The Nt-acetylation level of Rsa3 (S-) increased from 42% to 72%, whereas an increase from 12% to 44% was observed for Rpl7a (A-). In these cases, however, entry into stationary phase was associated with decreased protein levels (Fig. 6). Our shotgun proteomics data confirmed reduced abundance of Rpl7a (assigned to cluster 5). The lack of NatA-mediated Nt-acetylation caused a marked increase in Rpl7a and Rsa3 protein levels, the latter only during starvation.

Consistent with previous studies, the protein level of the core autophagy component Atg8 (MK-) increased during starvation (Fig. 6) (50). Simultaneously, the Nt-acetylation level of Atg8 increased from 26% to 65%. Loss of NatC activity did not, however, have a considerable effect on Atg8 protein levels. The largest variation in Nt-acetylation between exponential and stationary phase was observed for Smy2 (MK-) (*i.e.* from 34% to 100%). There are diverging reports on the cellular role of Smy2, including mRNA surveillance (51, 52), targeted translation control (53), and vesicular transport in the early secretory pathway (54, 55). The protein level of Smy2 decreased upon entry into stationary phase, an effect that appeared independently of NatC-activity. We also examined the expression of Pbp1 (MK-), a protein that localizes to cytoplasmic stress granules during glucose deprivation (56) and Tom70 (MK-), a member of the outer mitochondrial membrane protein complex (57). Both shotgun proteomics and immunoblot analysis showed decreased expression of Pbp1 in stationary phase, whereas no major effect was observed for Tom70. Loss of NatC-activity did not have any apparent effect on Pbp1 and Tom70 protein levels.

## DISCUSSION

The biological significance of Nt-acetylation is becoming increasingly evident, but still we know very little about the molecular mechanisms and the cellular effects of this widespread protein modification and whether it is subject to regulation. Our study revealed that yeast cells maintain global Nt-acetylation levels during unfavorable growth conditions. Indeed, we found that the steady-state levels of Nt-acetylation did not majorly fluctuate between active and stationary growth phases, despite a prominent decrease in acetyl-CoA levels. Overall, our data suggest that Nt-acetylation, in general, is not a very dynamic protein modification. Our findings contradict a previous study in mammalian cells proposing that Nt-acetylation is globally regulated by cellular metabolism and acetyl-CoA availability (58). The discrepancy between the two studies might be explained by differences in the experimental setups. To investigate possible metabolic regulation of Nt-acetylation, Yi and colleagues developed a biochemical method where subtiligase biotinylates unmodified protein N termini that are subsequently assessed by immunoblotting. This indirect method was employed on a limited set of apoptotic regulators, including several caspases (58). We instead performed untargeted N-terminal proteomics to investigate global changes in Nt-acetylation in response to changes in nutrient availability. In order to enrich for N termini the COFRADIC technology relies on extensive fractionation, which increases sensitivity but may simultaneously induce sample loss. Nevertheless, this experimental approach allowed us to obtain quantitative Nt-acetylation data and to cover a wider range of proteins as compared with the subtiligase-based method (58). In total, we observed 1,168 unique N termini accounting for 26.5% of the theoretically detectable proteome and 17.4% of the entire yeast proteome. Based on the observation that less than 1% of all measured Nt-acetylation values differed more than 10% when comparing the degree of Nt-acetylation between two independent control experiments (>1000 N termini) (3), we only analyzed one sample per time point. Thus, we cannot rule out the possibility that N-terminal peptides with biological functions remain undetected. It could also be that higher eukaryotes have evolved mechanisms to regulate Nt-acetylation and use it more prominently for regulatory purposes.

**Fig. 7.**
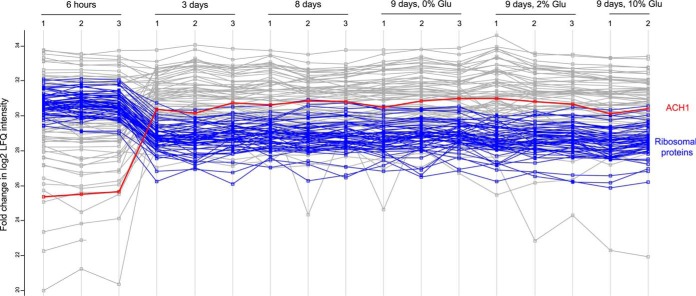
**Protein abundance varies during yeast growth.** Based on iBAQ values in all 17 samples analyzed by shotgun proteomics, a profile plot of the fold changes in LFQ intensities for the 100 most abundant proteins identified is shown. The profile of acetyl-CoA hydrolyze (Ach1), highlighted in red points to increased expression of Ach1 upon entry into the stationary phase. The profiles of 45 ribosomal proteins (highlighted in blue) are indicative of the generally decreased steady-state levels of ribosomal proteins upon entering stationary phase.

Although Nt-acetylation represents a considerable energy cost it remains stable despite major metabolic reprogramming. This finding emphasizes the importance of Nt-acetylation and indicates that the levels are there for a reason and is necessary for general protein function, regardless of metabolic state. From a metabolic perspective this raises several interesting questions, including how intertwined is mitochondrial and cytosolic acetyl-CoA metabolism and to what extent does NAT activity affect acetyl-CoA abundance? We found that the expression of acetyl-CoA hydrolase (Ach1) increased substantially following entry into stationary phase (Fig. 7 and supplemental Table S9). This finding is consistent with a previous study showing that the expression of *ACH1* is glucose-repressed (59). Ach1 was initially perceived as a mitochondrial acetyl-CoA hydrolase (60, 61). However, Fleck and Brock later suggested that Ach1 promotes acetate detoxification by transferring the CoA-moiety from succinyl-CoA to acetate. Thus, Ach1 would conserve energy rather than perform energy-wasting hydrolysis (62). According to Chen and colleagues these two processes are not mutually exclusive. They were able to identify a putative route for acetyl-CoA synthesis where Ach1 catalyzes the hydrolysis of intra-mitochondrial acetyl-CoA to acetate which is shuttled to the cytosol where it can be reconstituted to acetyl-CoA by Acs1/2 (63). Based on these findings, we hypothesize that during glucose exhaustion small quantities of mitochondrial acetyl-CoA could be transferred to the cytosol as acetate, which is subsequently reconstituted by cytosolic Acs1. Overall, this may contribute to a local accumulation of acetyl-CoA which is used by the NATs to cotranslationally acetylate nascent polypeptides. Histone acetylation would not be able to benefit from such a confined cytosolic increase because it is secluded in the nucleus. Monitoring the level of acetyl-CoA in different subcellular compartments and during various metabolic conditions is paramount in understanding how enzymes and other factors contribute to the cellular distribution of acetyl-CoA (24, 64). Unfortunately, these measurements are hampered by technical difficulties. Thus, whether the maintenance of global Nt-acetylation levels can be attributed to local pools of mitochondrial-derived acetyl-CoA, or perhaps is caused by a higher affinity of NATs *versus* KATs for acetyl-CoA, remains to be determined. The NatA complex is considered being the major contributor of Nt-acetylation and deletion of its catalytic subunit Naa10 does not appear to affect the intracellular level of acetyl-CoA, as determined by acetyl-CoA profiling (supplemental Table S3).

We also observed reduced levels of several ribosomal subunits following entry into stationary phase (Fig. 7 and supplemental Table S9), which is consistent with reduced translation rates at such conditions. This finding may partly explain why the protein level of most NAT subunits was reduced following nutrient starvation (Fig. 5), as the NAT enzymes act mainly cotranslationally. Despite the marked decline in acetyl-CoA levels and reduced NAT presence, most proteins maintained their Nt-acetylation status in exponential *versus* starvation-induced stationary phase. For instance, Naa40/NatD specifically acetylates histones H2A and H4 (65), and we found that H2A (Hta1) was fully Nt-acetylated under all conditions tested.

Nt-acetylation of annotated protein N termini is generally unaffected by the transition into stationary phase. We did, however, find several examples of dynamic Nt-acetylation where the proteins undergo significant changes in Nt-acetylation in response to changes in nutrient availability (Fig. 2, Table I). There are at least four possible scenarios that can account for this: (1) a regulated deacetylation of selected protein N termini, (2) specific degradation of N termini depending on their acetylation status, (3) *de novo* Nt-acetylation, or (4) altered translation rates. Since N-terminal deacetylases are yet to be discovered, Nt-acetylation is still considered to be an irreversible protein modification. Thus, this is not the most likely scenario. By clustering differentially acetylated N termini according to NAT specificity we found that these were evenly distributed among the NATs. This finding supports targeted degradation, rather than *de novo* acetylation, as the causative factor for the observed differential Nt-acetylation. That is, protein degradation affects the ratio of acetylated and unacetylated forms of specific proteins. The increased levels of Naa20 and Naa50 during starvation could potentially cause increased Nt-acetylation of their target substrates. However, this does not provide an overall explanation for the observed changes, like for the affected NatA substrates. Thus, other factors such as altered translation rates might also (indirectly) impact the degree of Nt-acetylation for a subset of proteins upon starvation. In this respect, it is noteworthy that alternative translational start site selection is subjected to regulations (66, 67), thereby changing the repertoire of Nt-proteoforms expressed. The ∼2-fold higher spectral counts observed for alternative N termini in the stationary setup hint to their increased abundance by increased synthesis. The combination of increased synthesis and reduced acetyl-CoA level might reduce the efficiency of Nt-acetylation, which could explain why the majority of affected alternative N termini were less acetylated in stationary phase.

Yeast stores two types of glucose polymers, namely glycogen and trehalose, and their quantities vary with environmental conditions (68, 69). Lillie and Pringle showed that both polymers accumulate in cells approaching stationary phase (70). Trehalose synthesis commences with glucose exhaustion, whereas glycogen synthesis is simultaneously shifted toward degradation. Glycogen synthase is partially regulated by the cyclin-dependent kinase Pho85. Deletion of the cyclin partners *PCL8* and *PCL10* results in activation of glycogen synthase and hyperaccumulation of glycogen (47). Our study revealed that Pcl8 (A-) is differentially Nt-acetylated between exponential and stationary phase (74% *versus* 100%). Moreover, deletion of *NAA10* prevented up-regulation of Pcl8 in stationary phase (Fig. 6). We suggest that NatA-mediated Nt-acetylation of Pcl8 is important for its stability and possibly its activity in the Pcl8-Pcl10-Pho85 complex. In this regard, it is also worth noting that Tps1 and Tps2, two enzymes involved in trehalose synthesis (69), and Pgi1, which catalyzes the interconversion of glucose-6-phosphate and fructose-6-phosphate, were also differentially Nt-acetylated.

Intriguingly, we found that two factors that are required for efficient ribosome biogenesis was also regulated by NatA (Fig. 6). The Nt-acetylation level of Rsa3 (S-) increased upon entry into stationary phase (from 42% to 72%). Moreover, lack of NatA activity resulted in increased Rsa3 protein levels during starvation. Rsa3 likely associates with pre-60S ribosomal particles and has a role in ribosome maturation (48, 71). Overexpression of Rsa3 improves growth during environmental stress (72). Conversely, deletion of *RSA3* leads to a deficit in free 60S ribosomal subunits, an accumulation of half-mer polyribosomes, and a slight reduction in the amount of mature 25S rRNA (48). Interestingly, similar phenotypes were observed in yeast lacking the ribosomal 60S subunit L7a (Rpl7a). Jakovljevic and colleagues showed that depletion of Rpl7a leads to defects in pre-rRNA processing and diminished levels of four neighboring ribosomal proteins (49). We found that the Nt-acetylation level of Rpl7a (A-) increased from 12% to 44% upon entry into stationary phase. Moreover, lack of NatA-mediated Nt-acetylation lead to increased Rpl7a protein levels. Both Rsa3 and Rpl7a are required for efficient pre-rRNA processing, as assembly factor and ribosome subunit respectively (48, 49, 71). The decreased protein levels of Rsa3 and Rpl7a observed in stationary phase complies with reduced ribosome biogenesis and cellular savings during less favorable conditions (73). Because a similar decline is not observed in yeast lacking *NAA10*, Nt-acetylation seems to be essential in this regard, perhaps through a destabilizing effect. Furthermore, Nt-acetylation of ribosomal proteins may be required for optimal protein synthesis. Kamita *et al.* reported decreased protein synthesis of ribosomes purified from NatA and NatB mutant strains. Moreover, they proposed that NatA has a role in translation fidelity whereas NatB is important for proper 80S ribosome assembly (74). Finally, we note that deletion of NatA subunits leads to slow growth, reduced glycogen accumulation, temperature sensitivity, defective mating, inability to enter stationary phase, and inability to sporulate, suggesting that NatA has an important role in determining cell fate (75–77).

In summary, our study suggests that nutrient starvation impacts Nt-acetylation of specific proteins. These acetylation events might be critical for the cellular adaptation to starvation, including regulation of glycogenesis and ribosome biogenesis. The herein observed fluctuations in Nt-acetylation may be connected to studies performed in *A. thaliana* (17) and *C. elegans* (18), in which Nt-acetylation was linked to various stress responses and further supports a role for Nt-acetylation in stress resistance (17, 18). Nt-acetylation is implicated in cardiac arrhythmia, developmental delay (20, 78–84) and cancers (85). Identifying factors that regulate Nt-acetylation is therefore of great importance. Hopefully, our findings will provide clues for further studies on the regulation of NAT activity and Nt-acetylation.

## DATA AVAILABILITY

The mass spectrometry proteomics data have been deposited to the ProteomeXchange Consortium (http://proteomecentral.proteomexchange.org) via the PRIDE partner repository (REF) under the identifiers PXD004326 and PXD009214. Annotated spectra have been made available in MS-Viewer via ProteinProspector (http://msviewer.ucsf.edu/prospector/cgi-bin/msform.cgi?form=msviewer) with the following search keys: 8i9cwelrjh (COFRADIC data) and ibi5m5495s (Shotgun data).

## Supplementary Material

supplemental Table S1

## Footnotes

^1^ The abbreviations used are:

Nt: N-terminal
Acetyl-CoA: acetyl coenzyme A
COFRADIC: COmbined FRActional DIagonal Chromatography
iMet: initiator methionine
KAT: lysine acetyltransferase
NAA/Naa: Nα-acetyltransferase gene/protein
NAT: N-terminal acetyltransferase
TAP: Tandem Affinity Purification.
