# Supplementary material for: N-terminal Acetylation Levels Are Maintained During Acetyl-CoA Deficiency in Saccharomyces cerevisiae
Source: Mol Cell Proteomics. 2018 Aug 27;17(12):2309–23. doi: 10.1074/mcp.RA118.000982 (PMC6283290; doi:10.1074/mcp.RA118.000982)
Supplement: supplemental Table S1 [file RA118.000982_index.html]

Supplement to N-terminal acetylation levels are maintained during acetyl-CoA deficiency in Saccharomyces cerevisiae | Molecular & Cellular Proteomics

## Supplemental Data

- Table S1 - Yeast strains used in this study.
- Table S2 - Primers used in this study.
- Table S3 - Acetyl-CoA profiling of wt and naa10&#x0394; yeast strains using MS.
- Table S4 to S6 - Table S4: Complete list of annotated and alternative N-termini identified in six different metabolic conditions and their N-terminal acetylation status. Table S5: List of Nt-acetylation affected annotated true N-termini Table S6: List of Nt-acetylation affected alternative N-termini.
- Tables S7 to S9 - Table S7: Complete list of 2,707 yeast proteins identified by shotgun proteomics using LC-MS/MS. Table S8: List of 1,918 quantified yeast proteins (quantified = proteins with at least 3 valid LFQ intensity values in one of the 6 conditions). Table S9: List of 401 significantly regulated proteins clustered after ANOVA analysis.
- Table S10 - Complete list of differentially acetylated N-termini with protein abundance data from shotgun analysis.
